# Is There a Relationship Between the Gut Microbiome and the Functional Capabilities of Skeletal Muscle? A Narrative Review

**DOI:** 10.3390/nu18193300

**Published:** 2026-10-08

**Authors:** Kristin S. Ondrak, Evan A. Seicshnaydre, Sharon J. Duffy, Corinna L. Rodriguez, Cassidy Scott, Sheri Gladish, Lauri O. Byerley

**Affiliations:** 1 Sports and Health Sciences, School of Health Sciences, American Public University System, Charles Town, WV 25414, USA; corinna.rodriguez@mycampus.apus.edu (C.L.R.); cassidy.scott0@mycampus.apus.edu (C.S.); sheri.gladish@mycampus.apus.edu (S.G.); lauri.byerley1@mycampus.apus.edu (L.O.B.); 2 Department of Exercise and Sport Science, University of North Carolina at Chapel Hill, Chapel Hill, NC 27599, USA; 3 Department of Physiology, Louisiana State University Health Sciences Center, New Orleans, LA 70112, USA; eseics@lsuhsc.edu; 4 Division of Libraries, Louisiana State University Health Sciences Center, New Orleans, LA 70112, USA; sduffy@lsuhsc.edu

**Keywords:** gut microbiome, diet-gut-muscle axis, skeletal muscle, muscle function, sarcopenia, short-chain fatty acids, exercise, probiotics

## Abstract

Diet is one of the primary determinants of gut microbiota composition and its metabolic output. That output is emerging as a critical determinant of systemic health and has been linked to most physiological systems in the body, yet its relationship to skeletal muscle physiology has only recently received attention. Evidence from experimental, observational, and clinical studies supports the existence of a gut–muscle axis, in which microbial composition and metabolic activity in the gut, influenced by diet, affect muscle metabolism, strength, function, and physical performance. Fiber, protein intake, probiotics, prebiotics, synbiotics, and fermented foods are examples of dietary constituents that transiently alter the gut microbiota, producing key metabolic mediators including short-chain fatty acids (SCFA), microbially generated amino acid products, and metabolites implicated in systemic inflammation and gut barrier integrity, alongside endocrine crosstalk involving myokines and hormones (e.g., cortisol and testosterone). Human studies consistently associate higher microbial diversity and enrichment of SCFA-producing taxa with improved muscle-related outcomes, while age-related dysbiosis has been linked to sarcopenia and functional decline. This narrative review summarizes current evidence of the gut–muscle axis and highlights potential mechanisms linking gut microbial composition to skeletal muscle function. Future advances in personalized nutrition may offer novel opportunities to preserve muscle health and functional capacity throughout aging.

## 1. Introduction

The gut microbiome and its metabolites have been shown to influence numerous organs, including the brain, liver, lung, skin, and heart, among others. The gut–brain axis was among the first to be identified more than 260 years ago [1], predating the microbiome sequencing boom. More recently, amid the microbiome research boom starting in the 2010s, the gut–muscle axis has been identified and investigated. A PubMed search of the term “gut-muscle” yielded 234 papers (3 July 2026), with the number of papers doubling beginning in 2023. According to PubMed, the first paper to use this term was published in 2017 [2].

The gut microbiome is a mixed community of several microbial types, including bacteria, archaea, fungi, viruses, and protozoa. Bacteria are the most abundant of these and have received the most attention. They are composed of thousands of different species, most of which fall into these major phyla: Firmicutes (reclassified as Bacillota), Bacteroidetes (Bacteroidota), Actinobacteria (Actinomycetota), Proteobacteria (Pseudomonadota), and Verrucomicrobia, which have been identified using 16s rRNA or shotgun metagenomics analysis. Although the relative abundance of the gut’s two predominant bacterial phyla, Bacillota and Bacteroidetes (90%), remains fairly constant, shifts are observed during aging and certain disease states [3]. The taxa within each phylum can shift dramatically under different conditions such as diet, sleep, physical activity, aging, disease, and antibiotic usage [3,4].

A symbiotic relationship exists between the human body and the bacteria that live on and in it, including in the gut. Bacteria respond to the conditions in which they grow and function, metabolizing the substrate presented in that environment and producing metabolites. As for the bacteria in the colon, they have been shown to produce metabolites that can influence skeletal muscle, both mass and function, which is the focus of this narrative review.

There are three types of muscle, smooth, cardiac and skeletal, of which skeletal muscle is critical for body movement. This review will focus on skeletal muscle, and both the quantity (mass) and quality (function) are critical for performance, depending on the task. Muscle mass, or quantity, is important for tasks that involve a great amount of force production, such as lifting heavy weights. Alternatively, muscle quality relates to the muscle’s functions, including strength, power, and the capacity to do work. However, muscle mass does not always align with function or quality. For example, the clinical definition of sarcopenia identifies muscle strength (function/quality), rather than mass, as the primary diagnostic criterion because strength correlates more closely with real-world outcomes such as falls, disability, and mortality [5]. Recent review articles have examined the relationship between the gut microbiome and skeletal muscle mass [6,7]. This narrative review will focus on skeletal muscle function and the gut microbiome. It highlights the microbiome, rather than specific microbiota, as a modifiable regulator of skeletal muscle function, mediating its effects through bioactive metabolites produced from dietary constituents that influence metabolic, immune, and endocrine signaling pathways.

## 2. Materials and Methods

For this narrative review, we searched PubMed, Google Scholar, and Embase for relevant peer-reviewed papers, with the final search occurring on 29 May 2026. We did not exclude any articles based on year of publication, although most papers on this topic have been published in the past decade, coinciding with advances in microbiome sequencing. Keywords, used individually or in combination with the Boolean operators, and included combinations of “gut barrier integrity”, “gut microbiome”, “gut microbiota”, “gastrointestinal microbiome”, “skeletal muscle”, “skeletal muscle metabolism”, “musculoskeletal health”, “muscle mass”, “muscle strength”, “muscle function”, “muscle recovery”, “physical activity”, “exercise interventions”, “sarcopenia”, “gut-muscle axis”, “short-chain fatty acids”, “myokines”, and related terms, along with specific terms such as “probiotics”, “prebiotics”, “fecal microbiota transplantation”, “cortisol”, and “testosterone.” Reference lists of key articles and existing reviews were also screened to identify additional relevant articles. Both animal and human studies were included to capture mechanistic and translational evidence, and article types included original research, systematic reviews, meta-analyses, and other narrative reviews. Non-peer-reviewed sources, conference abstracts, and articles not published in English were excluded. Given the narrative, rather than systematic, design of this review, article selection was based on relevance to the topics covered in each section rather than on a predefined, reproducible screening protocol, and the search was not exhaustive. This approach may be subject to selection bias, and the number of eligible articles identified via database searches versus reference-list screening was not formally tracked.

The ideas, structure, and final content were developed by the authors, who controlled the content throughout the process. ChatGPT 5.6 (OpenAI, 2026) was used to develop an initial outline. Searches for articles were conducted using PubMed, Google Scholar, and Embase. Claude (Claude-Sonnet-4-6, large language model, Anthropic, San Francisco, CA, USA) was used to locate articles and evidence that PubMed, Google Scholar, and Embase searches may have missed. Mendeley (New York City, NY, USA) was used to organize research and insert proper citations in several rough drafts of the text. EndNote (Clarivate, Philadelphia, PA, USA) was used for the final organization and insertion of references. Grammarly (San Francisco, CA, USA) was used to check for correct citations, plagiarism, and clarity. The figures were drawn with the assistance of Claude (Anthropic, San Francisco, CA, USA) and BioRender (Biorender.com, Toronto, ON, Canada).

## 3. Skeletal Muscle Physiology and Function

The primary functions of skeletal muscle are to produce force and cause movement, but it also plays an important role in storing energy. Amino acids produced during protein breakdown and adaptation are stored in skeletal muscle, as is glycogen produced during anabolic processes. Having these energy reserves readily available in skeletal muscle allows for quick access to ATP needed for force production, rather than waiting for energy to arrive from the bloodstream. In fact, muscle glycogen stores increase following aerobic training [8] when appropriate dietary sources of carbohydrates are provided [9].

The functions of skeletal muscle are influenced by factors such as age, exercise and disease states. As humans age, the properties of skeletal muscles change, and sarcopenia develops [10]. Sarcopenia is the age-related, progressive loss of skeletal muscle mass, strength, and physical function. This age-related decline in muscle mass occurs at a rate of ~1 to 2% per year after the age of 50, which in turn leads to a reduction in force production capabilities and an impaired ability to rebuild damaged muscle [11]. In addition to the loss of muscle mass, the distribution of muscle fiber types also changes with age. Muscle biopsies show a reduction in type IIa and IIb fibers with age and an increase in the percentage of type I fibers [12]. These age-related shifts favor lower-intensity, longer-duration activities in lieu of higher-intensity, explosive movements.

While age has negative effects on muscle, exercise is extremely beneficial and can counteract age-related declines. These benefits include hypertrophy, enhanced mitochondrial activity, increased capillary density and vasodilation, improved metabolic efficiency, and muscle function [13,14,15]. It is well established that endurance training leads to increased muscle volume and power in older adults [16], as well as mitochondrial enzyme activity [17,18,19] and ultimately to the production of ATP and aerobic capacity. On the other hand, resistance training has been shown to improve muscle mass and strength [20,21].

Finally, it is important to discuss how muscle quality is measured. Muscle quality is defined as the ratio of muscle strength relative to mass, and common measurements include grip strength to indicate neuromuscular function, metabolic measures such as mitochondrial density, muscle fiber typing, and functional performance tests. Grip strength measured via dynamometry has been shown to be a reliable indicator of muscle quality in older adults [22] and, not surprisingly, decreases with advancing age [23]. Mitochondrial density and function, on the other hand, require invasive muscle biopsy measurements and are therefore more difficult to obtain, despite being the gold standard. Less invasive measurements of muscle metabolic activity include oxygen consumption during submaximal and maximal exercise tests, PET-CT scans, and blood flow perfusion techniques. Functional performance tests to quantify muscle function include the sit-to-stand test or the 6 min walk test. The gold standard for measuring muscular strength, on the other hand, is isokinetic dynamometry. These functional tests are relatively simple to administer and provide valuable information about muscular function and cardiometabolic health.

## 4. Mechanistic Links Between the Gut Microbiome and Skeletal Muscle

**Figure 1:**
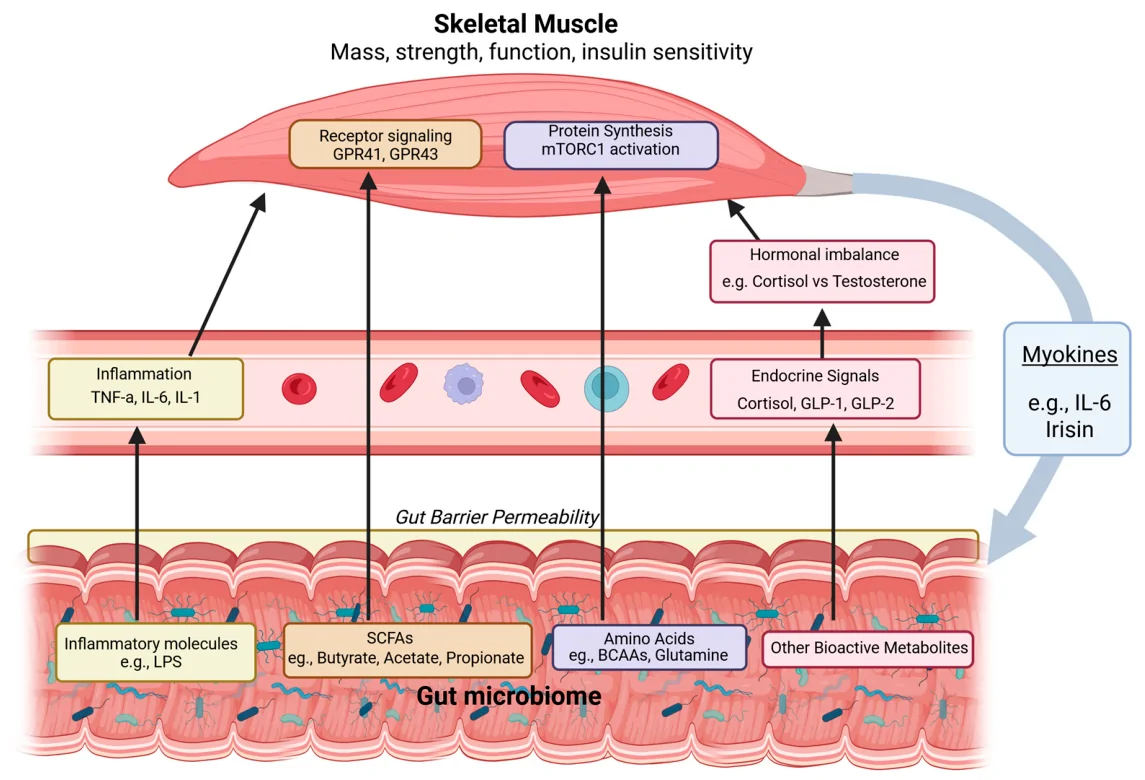
Demonstrates the crosstalk between the gut microbiome and skeletal muscle that influences skeletal muscle function. The gut produces beneficial microbial metabolites, particularly short-chain fatty acids (SCFAs), which contribute to improved mitochondrial function, insulin sensitivity, immune regulation, gut barrier integrity, and endocrine signaling in the skeletal muscle. Conversely, gut dysbiosis may promote intestinal permeability, lipopolysaccharide (LPS) translocation, systemic inflammation, and anabolic resistance, resulting in muscle dysfunction. Skeletal muscle produces several myokines, such as Il-6 and irisin, that affect the gut microbiome. Created in BioRender, Lauri O. Byerley (2026), https://BioRender.com.

The gut microbiome is linked to skeletal muscle mass, strength, and function through several pathways. An important one is the metabolites produced from undigested dietary substances that reach it. Table 1 shows metabolites produced by the gut microbiome that affect skeletal muscle function. We have focused on metabolites for which evidence is strongest. Figure 1 provides an overview of these metabolites and their routes of action.

### 4.1. Dietary Fiber and Short-Chain Fatty Acid Production

When gut microbiota ferment dietary fiber, they produce short-chain fatty acids (SCFAs), primarily acetate, propionate, and butyrate, that act as both energy substrates and signaling molecules for skeletal muscle [29]. Acetate enhances mitochondrial function and glucose metabolism in myocytes, while butyrate helps preserve muscle mass by suppressing inflammation and excessive autophagy, in part by activating the AMPK–PGC-1α pathway, which drives mitochondrial biogenesis [29]. This relationship has direct human evidence, as research has shown that gut microbial synthesis of butyrate was positively associated with skeletal muscle mass in healthy menopausal women, supporting a role for SCFA production in muscle maintenance rather than a mere correlation [24,30].

When the gut barrier is compromised, it can promote systemic inflammation. It is well known that a low-fiber diet erodes the colonic mucus barrier, increasing intestinal permeability [31]. When this occurs, bacterial lipopolysaccharide (LPS) can leak into circulation, producing a state termed “metabolic endotoxemia.” This was first causally demonstrated by Cani et al. (2007) [32], who showed that experimentally raising plasma LPS in mice was sufficient to induce insulin resistance and adiposity independent of diet. Because skeletal muscle is the primary site of insulin-stimulated glucose disposal, LPS-driven inflammation impairs insulin signaling in myocytes, reducing glucose uptake and contributing to insulin resistance [32,33]. This mechanism has functional consequences as it impacts the relationship between exercise and metabolic health. In a human interventional study of 39 men with prediabetes, Liu et al. (2020) [34] found that the degree to which an individual’s gut microbiota could ferment dietary substrates into beneficial metabolites predicted how effectively exercise improved their glucose homeostasis and insulin sensitivity, with “responders” showing enhanced short-chain fatty acid biosynthesis compared to “non-responders.” This suggests the microbiome does not simply respond to metabolic disease but actively modulates an individual’s response to exercise as a metabolic intervention. Taken together, these findings position gut-derived inflammation as a mechanistic bridge connecting dysbiosis, impaired muscle insulin sensitivity, and downstream declines in muscle metabolic efficiency.

At the molecular level, the link between gut-derived inflammation and impaired muscle insulin sensitivity is well characterized. Circulating pro-inflammatory cytokines such as TNF-α activate stress kinases, including c-Jun N-terminal kinase (JNK) and the IKKβ/NF-κB pathway, both of which converge on insulin receptor substrate-1 (IRS-1), driving inhibitory serine phosphorylation that disrupts normal insulin signal transduction [35,36,37]. This impairment has been demonstrated in humans. Plomgaard et al. (2005) [33] infused healthy volunteers with TNF-α and showed that it induced measurable insulin resistance in skeletal muscle, mediated by an inhibition of phosphorylation of Akt substrate 160, a critical step required for GLUT4 glucose transporter translocation to the muscle cell membrane. Because LPS-driven gut permeability is one of the primary physiological triggers of elevated circulating TNF-α [32], this pathway offers a plausible mechanistic chain connecting intestinal dysbiosis to reduced glucose uptake and metabolic efficiency in muscle tissue, independent of any direct effect on muscle mass.

### 4.2. Dietary Protein

Dietary protein acts directly as an anabolic stimulus for skeletal muscle and an independent modulator of gut microbial composition through putrefaction. Most dietary protein is absorbed in the small intestine, allowing it to act directly on skeletal muscle, which is not the focus of this review. The protein/peptides/amino acids that are not absorbed in the small intestine travel to the colon, where they undergo putrefaction. Protein quantity and source both matter, producing distinct gut microbiota patterns. Human cohort data reinforce this dose- and source-dependence, showing that both the quantity and vegetable/animal ratio of habitual protein intake are associated with distinct gut microbiome profiles in older adults [38]. At the mechanistic level, dietary protein is metabolized by proteases and peptidases in the small intestine, and amino acids that escape absorption can be used for protein synthesis by gut microbes, meaning the balance and amount of dietary macronutrients directly shape intestinal microbiota composition [39].

The gut microbiota also contributes directly to the host’s amino acid economy by producing or breaking down amino acids. Human isotope tracer studies have shown that a meaningful fraction of the body’s circulating essential amino acid pool comes from gut bacterial synthesis, not just dietary intake. Specifically, approximately 2–20% of circulating lysine in human plasma, urine, and body proteins has been shown to originate from the gut microbiota using isotope tracers in humans, pigs, and rats [40]. Separately, gut microbiota-derived lysine and threonine have been shown to be important contributors to the free lysine and threonine pool in humans fed nitrogen-adequate diets [41]. Newsome et al. (2020) [42] demonstrated that anywhere from 5% (threonine, lysine, and phenylalanine) to approximately 60% (valine) of specific microbially synthesized essential amino acids, depending on dietary protein content, were incorporated into skeletal muscle tissue in mouse models. Accordingly, researchers found that 7.3 to 25.7% of host muscle essential amino acids were of microbial origin in deer mice [43].

Certain bacterial taxa, including *Lactobacillus* and *Bifidobacterium* species, synthesize amino acids de novo, thereby increasing their systemic availability, particularly under conditions of dietary protein restriction [44]. This is especially relevant to aging muscle, where reduced microbial diversity and altered SCFA/amino acid output have been proposed as contributors to “anabolic resistance,” or the blunted muscle protein synthesis response to protein intake present in older adults. These observations link changes in gut composition to the age-related decline in muscle mass described in research on dietary protein and sarcopenia [44,45].

Most dietary protein digestion and absorption happens in the small intestine, but anywhere from 3 to 18 g/day can reach the colon [46], where the gut bacteria putrefy it, producing products such as ammonia, hydrogen sulfide, branched-chain fatty acids, and p-cresol, several of which have been shown to increase intestinal inflammatory response and tissue permeability rather than protect against it [47]. One such metabolite, imidazole propionate, derived from histidine, has been shown to impair insulin signaling by activating the same mTORC1 pathway responsible for amino acid-driven muscle protein synthesis, suggesting that bacterial amino acid metabolism can undermine muscle metabolic health through the very pathway it otherwise supports [48].

### 4.3. Other Bioactive Molecules

Fruits, vegetables, tea, and other plant-based foods contain dietary polyphenols, a distinct nutrient class whose muscle-relevant effects appear to be substantially mediated by gut microbial biotransformation. In the gut lumen, gut microbiota extensively biotransform dietary polyphenols into a wide range of bioactive compounds that affect skeletal muscle. Beneficial effects may vary across individuals depending on the composition and metabolic function of their gut bacterial communities. This microbiota-dependent bioactivation has been most clearly demonstrated for compounds such as urolithins (from ellagitannins) and specific flavonoid subclasses, thereby positioning polyphenol-microbiota interactions as a mechanistic bridge between diet and its myoprotective effects [49].

## 5. Immune System Modulation

The immune system communicates between the intestinal environment and peripheral tissues, influencing the body’s inflammatory and metabolic responses. Alterations in gut microbiota can influence systemic immune tone, which in turn affects muscle homeostasis, recovery, and adaptation. Gaining a deeper understanding of this immune-mediated connection provides valuable insight into how gut health contributes to overall physical performance and the prevention of muscle deterioration.

### 5.1. Gut Barrier Integrity and Systemic Inflammation

The gut barrier serves as a critical interface between the external environment and the host’s internal systems. When it functions properly, it allows nutrients and other small molecules to be absorbed while also restricting the passage of harmful substances. Disruptions in the gut microbial balance often result from poor diet, alcohol use, antibiotic exposure, or aging, all of which can compromise the protective barrier and lead to a condition often referred to as “leaky gut.” This allows bacterial fragments such as lipopolysaccharides (LPS) to enter the circulation, where they can initiate systemic immune activation. The dietary group polyphenols can help improve barrier integrity [50].

Once LPS molecules enter circulation, they bind to toll-like receptor 4 (TLR4) on immune cells. This interaction triggers the NF-κB signaling cascade, leading to the production of inflammatory cytokines such as tumor necrosis factor-α (TNF-α), interleukin-6 (IL-6), and interleukin-1β (IL-1β), which collectively drive a systemic immune response. However, SCFAs, particularly butyrate, can counteract this inflammatory process. Researchers have described how butyrate helps control inflammation by promoting the growth of regulatory T cells, which are activated through the PPAR-γ pathway [49]. This process blocks inflammatory signaling, including the NF-ĸB pathway, since it lowers the activity of the enzymes that promote inflammation. This regulatory action highlights how microbial metabolites influence immune signaling pathways that, in turn, affect muscle metabolism and function. When butyrate production decreases, it is often a result of dysbiosis. This weakens the protective mechanisms and allows persistent inflammation to occur, impairing muscle recovery and performance.

Human studies show that disruptions in gut microbial balance are often linked to higher levels of systemic inflammation and reduced muscle performance compared to healthy individuals [51]. This supports the idea that maintaining microbial diversity and gut barrier integrity is essential for regulating immune activity. When the gut barrier remains intact, it helps the body limit inflammatory responses that can negatively affect skeletal muscle metabolism. Therefore, maintaining a healthy, balanced microbiome plays a vital role in controlling inflammation and supporting optimal skeletal muscle function.

### 5.2. Inflammatory Pathways and Muscle Functions

Emerging evidence points to a connection between the gut and skeletal muscle, suggesting that inflammation may be one pathway through which the microbiome influences muscle metabolism. When the intestinal barrier becomes compromised, harmful bacterial fragments enter the bloodstream and trigger the release of proinflammatory cytokines. The gastrointestinal system does more than digest and absorb food; it also serves as a home to trillions of microbes that continuously adapt to factors such as diet, lifestyle, and overall health [52]. These microbes are beneficial because they help regulate major metabolic organs, including the pancreas, adipose tissues, and skeletal muscle [53]. This connection explains how changes in gut composition and barrier integrity can influence inflammation in other tissues. Chew et al. (2022) [52] suggested that when the gut loses anti-inflammatory bacteria, the intestinal barrier weakens, setting off a chain reaction of inflammation. This process increases the release of cytokines such as TNF-α and IL-6, which can negatively affect muscle mass and overall muscle function. Once in circulation, these cytokines, particularly TNF-α and IL-1, act directly on skeletal muscle, activating pathways that inhibit anabolic signaling and promote protein degradation through the ubiquitin-proteasome and autophagic–lysosomal systems [54,55]. This ongoing inflammatory activity interferes with muscle repair, contributes to muscle wasting, and reduces metabolic efficiency. Prolonged IL-6 exposure, distinct from the short-term rise after exercise, can increase oxidative stress and insulin resistance in muscle cells, impairing recovery and performance [56].

### 5.3. Chronic Inflammation and Sarcopenia

Building on evidence that inflammatory pathways contribute to muscle impairment, recent research demonstrates the link between gut dysbiosis and the chronic inflammation observed in sarcopenia. Wang (2025) [57] explained that the gut bacteria *Prevotella* and Bacteroidetes are responsible for fermenting dietary fiber and producing SCFAs, which play an important role in maintaining muscle mass and strength. When the ratio of these bacteria decreases, the body’s ability to produce SCFAs also declines, which can negatively affect muscle health. The study also found that lower levels of *Faecalibacterium* and Lachnospiraceae, two major producers of SCFAs, are associated with reduced SCFA concentrations, thereby increasing the risk of muscle atrophy and decreasing muscle performance. This evidence supports the notion that microbial shifts in the gut, particularly the loss of SCFA-producing species, contribute to a proinflammatory state that disrupts epithelial integrity. As cytokine activity increases, this sustained inflammation accelerates the loss of muscle mass and function, reinforcing the biological connection between gut imbalance and skeletal muscle deterioration.

Research has also shown that gut bacteria such as *Faecalibacterium prausnitzii* and *Bifidobacterium longum* support healthy aging and muscle function by producing SCFAs [58]. The study also noted that *F. prausnitzii* is associated with improved insulin sensitivity, balanced anabolic activity, and reduced inflammation, all of which help maintain muscle mass and overall function.

As the gut microbiota loses diversity and the balance between pro- and anti-inflammatory species shifts, it becomes more vulnerable to chronic inflammatory signaling. Over time, this persistent inflammatory response not only contributes to anabolic resistance but also accelerates muscle deterioration associated with aging. As people age, they experience a decline in skeletal muscle mass and strength, which alters their physical performance. The study described sarcopenia as a progressive condition that is characterized by the loss of lean muscle mass, strength, and function, leading to an increased risk of falls, disability, and other health conditions over time [58].

Chronic inflammation is one of the strongest mechanisms linking gut dysbiosis and skeletal muscle dysfunction. This directly supports the idea that the gut microbiome may play a role in regulating muscle performance. When microbial balance is lost, immune activation alters cellular metabolism, weakening the muscle’s ability to recover and adapt. Although anti-inflammatory medications such as corticosteroids and NSAIDs can provide short-term relief, their long-term use may further disrupt the composition of the gut microbiota and compromise epithelial integrity. In contrast, therapeutic approaches such as probiotic or prebiotic supplementation and diet modification can help restore microbial diversity and reduce inflammation, thereby supporting muscle health. Understanding the interaction between chronic inflammation, medication use, and microbial balance has meaningful implications for preventing muscle loss and promoting healthier aging.

## 6. Endocrine Signaling and Neuromuscular Impact

Beyond its direct metabolic contributions, the gut microbiome also shapes skeletal muscle health through endocrine and neuromuscular signaling pathways. Muscle itself functions as a secretory organ, releasing myokines that mediate crosstalk with the gut, while circulating hormones such as testosterone and cortisol, both of which are subject to microbial influence, regulate the anabolic–catabolic balance that determines muscle mass and function [59]. SCFAs, already discussed for their metabolic role, further intersect with this signaling network by modulating hormone secretion and neuromuscular signaling pathways relevant to muscle maintenance. Together, these interconnected endocrine mechanisms offer additional insight into how gut microbial composition may influence muscle physiology beyond the direct metabolic and inflammatory pathways discussed above.

### 6.1. Myokines

Myokines are cytokine and peptide signaling molecules secreted by skeletal muscle cells, or myotubes, particularly during exercise, and represent a key element of the gut–muscle axis, necessary for regulating energy metabolism, appetite, and overall health [59]. Their release demonstrates the muscle’s role as an endocrine tissue engaged in a highly integrated, communicative process with the gut microbiome [60]. This relationship is bidirectional: intestinal short-chain fatty acids (SCFAs), gut-derived metabolites produced by microbial fermentation, modulate myokine expression, while myokines and their downstream signals can, in turn, reshape gut microbial composition and metabolite output [61]. This reciprocal signaling loop positions the gut microbiome not as a passive bystander but as an active regulator of the muscle-derived endocrine signals discussed below.

Individual myokines exert distinct effects within this signaling network. For example, IL-6 stimulates intestinal L cells to secrete glucagon-like peptide-1 (GLP-1), a gut hormone that regulates appetite and energy metabolism, illustrating direct muscle-to-gut communication [59]. The gut-derived hormones GLP-1 and GLP-2 have also been shown to exert anti-sarcopenic effects by inhibiting excessive muscle protein breakdown and minimizing atrophy [42]. A third example, irisin, is cleaved from the membrane protein FNDC5 and released from muscle during exercise; beyond its established role in adipose tissue browning, irisin also appears to shape gut microbial composition directly, as irisin-deficient mice exhibit reduced Firmicutes and increased Bacteroidota populations along with lower abundances of *Lactobacillus* and *Bifidobacterium* [62]. Together, IL-6, GLP-1, GLP-2, and irisin illustrate how muscle-derived signals feed back into gut hormone secretion and microbial community structure, reinforcing the gut microbiome’s role as both a driver and a target of the gut–muscle axis.

A separate class of myokines acts locally to regulate muscle growth itself, and the gut microbiome appears to influence this pathway as well. Myostatin, a member of the transforming growth factor-β (TGF-β) family, is a key negative regulator of muscle mass; it inhibits skeletal muscle growth by binding to the activin type 2B receptor and activating Smad (mothers against decapentaplegic homolog) transcription factors, thereby promoting muscle wasting. Follistatin acts as myostatin’s principal antagonist, directly counteracting its effects to promote muscle growth and reduce atrophy. Gut microbiota-derived butyrate has been shown to counteract muscle atrophy in cachexia models by modulating intestinal barrier function and macrophage polarization [63], suggesting that microbial metabolites may intersect with the same atrophy-related pathways that myostatin and follistatin regulate locally. Considered alongside the metabolic myokines, this suggests the muscle-secreted signaling cascade is shaped by the gut microbiome at both the systemic and local level.

### 6.2. Testosterone

Testosterone production stimulates muscle protein synthesis and maintains neuromuscular junctions, necessary for muscle hypertrophy, repair, and maximum efficiency. It also affects psychological well-being and brain function. Studies have revealed differences in microbiota composition between males and females, largely attributable to differences in testosterone levels [64]. Evidence suggests that testosterone plays an important part in shaping the diversity of the gut microbiome, which, in turn, affects testosterone levels [65]. Research has shown that testosterone modulates microbiome diversity and community composition, although higher testosterone levels may be associated with reduced microbial diversity [66]. Alternatively, other data show that men with higher testosterone levels exhibit more diverse microbiome composition than those with lower levels [67]. Specific microbes, such as *Ruminococcus*, *Acinetobacter*, *Dorea*, and *Megamonas*, are positively associated with testosterone levels [68]. The gut microbiome itself may influence testosterone production; the hypothalamic–pituitary–gonadal (HPG) axis undergoes modulation that may affect the release of gonadotropin-releasing hormone (GnRH) and luteinizing hormone (LH), both of which stimulate testosterone synthesis [67].

### 6.3. Cortisol

Generally, psychosocial or psychological stress triggers cortisol secretion through the hypothalamic–pituitary–adrenal (HPA) axis. This relationship with the gut microbiome is bidirectional, such that elevated cortisol alters intestinal permeability and gut microbiota composition, while gut microbes influence HPA axis reactivity and the magnitude of the stress response. This forms a self-reinforcing cycle between stress physiology and microbial composition [69]. At the level of the muscle, cortisol directly inhibits protein synthesis and promotes muscle protein breakdown by activating the ubiquitin-proteasome and autophagy-lysosome systems, in part through upregulation of the atrophy-related genes atrogin-1 and MuRF-1 [70]. Cortisol also counteracts the effects of anabolic hormones, including testosterone, further tilting the balance toward muscle catabolism [71]. Because gut dysbiosis increases intestinal permeability and promotes the same LPS-driven systemic inflammation discussed earlier in this paper, chronic stress-induced dysbiosis may compound cortisol’s direct catabolic effects on muscle, creating overlapping pathways through which stress physiology and the gut microbiome jointly undermine muscle mass and function.

### 6.4. Short-Chain Fatty Acids

SCFAs are metabolites created in the colon when bacteria ferment dietary fiber [30]. There is a positive association between skeletal muscle and serum butyrate concentrations, as numerous analyses have demonstrated a relationship between microbial butyrate synthesis and appendicular lean mass [72]. Furthermore, *Faecalibacterium prausnitzii* and *Butyricimonas virosa*, two butyrate-producing bacteria, were positively associated with both microbial butyrate synthesis capacity (*F. prausnitzii:* Spearman correlation coefficient (SCC) = 0.25; *B. virosa*: SCC = 0.15 and skeletal muscle index (*F. prausnitzii*: SCC = 0.16; *B. virosa*: SCC = 0.17 [30]. These correlations, while statistically significant, were modest in magnitude, and a one-sample Mendelian randomization analysis in the same study provided evidence of an effect of gut microbial butyrate synthesis on appendicular lean mass (β = 0.04, 95% CI: 0.029–0.051, *p* = 0.003), suggesting a potential mechanistic relationship. Acting as both metabolic substrates and signaling molecules, SCFAs bind directly to G-protein-coupled receptors expressed on skeletal muscle cells themselves, including GPR41 (FFAR3) and GPR43 (FFAR2), triggering intracellular calcium signaling that promotes oxidative muscle fiber proliferation and enhances insulin-stimulated glucose uptake in myotubes [73].

SCFAs play a key role in mediating communication between the gut microbiome and skeletal muscle. Because skeletal muscles express SCFA-sensitive receptors (GPR41 (FFAR3), GPR43 (FFAR2), and GPR109A), they can directly regulate muscle metabolism and gene expression [30]. A cell culture study demonstrated that acetic acid directly stimulates GPR43 in L6 muscle myotube cells, triggering an intracellular calcium signaling cascade that activates calcineurin and drives the proliferation of slow-twitch (oxidative) muscle fibers, a direct, receptor-mediated effect entirely local to the muscle cell [74]. Separately, propionate and valerate were shown to increase insulin-stimulated glucose uptake in C2C12 skeletal muscle cells via GPR41, an effect that disappeared when GPR41 was silenced with siRNA. This demonstrates a direct receptor-level mechanism within the muscle, with no CNS involvement required [75]. SCFAs are crucial for protein synthesis; however, if SCFA production is limited in the gut, there is an increased risk of chronic inflammation, which often results in gradual muscle deterioration [72].

Evidence suggests strong interconnectivity between the gut microbiome, the central neuroendocrine system, and skeletal muscle, operating through several converging pathways. Muscle-derived myokines regulate gut hormone secretion and vice versa [59]; microbial modulation of the hypothalamic–pituitary–adrenal axis and its downstream effects on cortisol-driven muscle catabolism [69,70]; and short-chain fatty acids influence hormone signaling and muscle protein metabolism directly [44]. However, additional research is needed to fully understand the mechanistic links at the endocrine level. Much of the existing evidence is derived from animal models, particularly for pathways such as SCFA-mediated hormone modulation and microbiota-driven changes in HPA axis reactivity, and it remains unclear how directly these mechanisms translate to human physiology. Additionally, most evidence from human studies is correlational in nature, making it difficult to establish whether gut microbial composition drives observed endocrine and muscular changes or vice versa. Longitudinal studies that directly manipulate the gut microbiome, such as through targeted probiotic, prebiotic, or fecal microbiota transplantation interventions, while simultaneously tracking hormone levels (cortisol, testosterone, myokine profiles) and muscle mass or function outcomes, would help clarify these relationships. Similarly, the specific receptor-level mechanisms by which microbial metabolites interact with endocrine signaling pathways in muscle tissue remain incompletely characterized and represent a clear direction for future mechanistic work.

## 7. Evidence from Human Studies

While the mechanistic pathways described in this article outline how the gut microbiome influences skeletal muscle, the strength of this evidence varies considerably across study designs. This section first examines human observational studies, which identify associations between microbiome features and muscle outcomes, and then human interventional studies, which more directly test whether modifying the gut microbiome or its metabolites, or perturbing it through exercise, produces measurable changes in the microbiome, muscle outcomes, or both. Table 2 summarizes representative observational and interventional studies.

### 7.1. Cross-Sectional/Observational Studies in Humans

#### 7.1.1. Associations Between Microbiome Diversity and Muscle Mass/Function

Human observational studies have provided important evidence linking gut microbiome composition to skeletal muscle mass, offering support for the existence of a gut–muscle axis. Early research proposed that the gut microbiota contributes to age-related declines in muscle size, composition, and function, establishing a biological rationale for exploring this relationship further [87]. Building on this, a study of 1134 healthy menopausal women found a positive correlation between the gut microbiome’s capacity to synthesize butyrate and appendicular lean mass. Two butyrate-producing bacteria, *Faecalibacterium prausnitzii* and *Butyricimonas virosa*, were specifically associated with higher muscle mass markers, suggesting that the short-chain fatty acid-producing capacity of the microbiome plays a role in maintaining lean mass in this population [30].

Larger population-based studies have strengthened these associations. In the HUNT cohort study (n = 5196), three bacterial species, *Coprococcus comes*, *Dorea longicatena*, and *Eubacterium ventriosum*, were reproducibly associated with higher appendicular lean mass and hip bone mineral density across independent samples [76]. Participants who harbored all three species had an estimated 0.80 kg greater appendicular lean mass compared to those with none of the three, which is a clinically meaningful difference in the context of age-related muscle loss. A systematic review of twelve studies in adults aged 50 and older also found that overall gut microbiome diversity, or alpha diversity, was positively associated with muscle mass in three of four studies and with muscle strength in two of three studies, indicating that microbial richness itself may be relevant to muscle health beyond any single taxon [51]. These associations are not limited to muscle mass alone, as research has also linked gut microbiome composition to broader indicators of muscle function, including grip strength and physical performance, suggesting that the microbiome’s influence extends to both the quantity and quality of skeletal muscle [25].

However, not all findings point in the same direction. In the Wakayama study, a cross-sectional investigation of 744 community-dwelling Japanese adults aged 65 to 89, a higher abundance of the genus *Blautia* was significantly associated with lower appendicular skeletal muscle mass index, with *Blautia* being more abundant in the low-muscle-mass group [77]. This is somewhat counterintuitive given that other studies have found *Blautia* to be enriched in physically active individuals compared to sedentary ones [88,89]. This discrepancy likely reflects the fact that the relationship between specific microbial taxa and muscle outcomes is context-dependent, influenced by factors such as age, diet, and the broader composition of the gut microbiome. This finding highlights the importance of considering population characteristics when interpreting microbiome-muscle associations from cross-sectional data.

#### 7.1.2. Microbiome Profiles Across Varying Physical Activity Levels

Studies comparing the gut microbiomes of athletes and sedentary individuals have consistently identified differences in microbial composition, and several have connected these differences to muscle-related outcomes. Using accelerometer-verified activity data, Bressa et al. (2017) [88] found that active women had significantly higher abundances of *Blautia*, *Coprococcus*, and *Ruminococcus* compared to their sedentary counterparts. The abundances of *Blautia* and *Coprococcus* in the active group were also positively correlated with appendicular muscle mass, which represents one of the earlier studies to directly link an exercise-associated microbiome profile to a lean mass measure in humans. These taxon-level findings have been corroborated across a broader literature, with a systematic review of 38 human studies confirming that enrichment of *Coprococcus*, *Ruminococcaceae*, and Lachnospiraceae in physically active individuals was one of the most consistently replicated observations across different populations and study designs [90].

Research has also shown that the amount of exercise appears to matter, not just whether someone is active. Among 33 competitive cyclists, *Prevotella* abundance was significantly correlated with weekly training hours (*p* = 0.0026), and those training more than 11 h per week were substantially more likely to have high *Prevotella* levels than those who trained less [91]. This relationship was supported on a much larger scale in the SCAPIS cohort, where accelerometer-measured physical activity data from 8416 participants showed that *Prevotella copri* had strong positive associations with moderate-to-vigorous intensity physical activity and inverse associations with sedentary time [92]. Metagenomic analysis of 418 fecal samples from athletes, moderately active individuals, and sedentary adults further identified two distinct microbial clusters: one enriched in athletes and characterized by short-chain fatty acid-producing taxa within the *Blautia* and *Coprococcus* clades, and one enriched in sedentary individuals with comparatively less fermentative activity [89]. These studies together suggest that regular physical activity is associated with a gut microbiome profile that is richer in metabolically active, short-chain fatty acid-producing bacteria.

#### 7.1.3. Age-Related Alterations in Gut Microbiota and Their Association with Sarcopenia

The gut microbiome undergoes significant changes with age that appear to parallel the decline in skeletal muscle health. Research has shown that aging is associated with reduced microbial diversity, loss of beneficial short-chain fatty acid-producing bacteria, and increased gut permeability, all of which can contribute to the chronic systemic inflammation associated with muscle deterioration [93]. More specifically, levels of *Faecalibacterium*, Lachnospiraceae, and other fermentative genera tend to decrease over the lifespan while potentially harmful taxa expand in their place, shifting the gut environment toward a more proinflammatory state [94].

When researchers have examined individuals with sarcopenia specifically, these microbiome differences become more apparent. A systematic review of 17 studies found that reduced levels of *Faecalibacterium prausnitzii* and *Bifidobacterium longum* were among the most consistently reported findings in older adults with poor muscle mass or physical function, both of which are known to support gut barrier integrity and produce anti-inflammatory butyrate [58]. A meta-analysis focused specifically on sarcopenic individuals confirmed lower abundances of *Prevotella* and Lachnospiraceae alongside higher levels of Bacteroides and other proteolytic bacteria, and the reduction in *Faecalibacterium prausnitzii* emerged as one of the most replicated microbiome findings in this population [57]. Importantly, these microbiome changes in aging populations have been associated not only with losses in muscle mass but also with declines in physical performance measures such as grip strength and gait speed, indicating that gut dysbiosis may contribute to the full clinical picture of sarcopenia rather than muscle atrophy alone [95].

Reduced overall microbial diversity has also been associated with worse muscle parameters in older adults across multiple studies. A systematic review of twelve studies in adults aged 50 and older found inconsistent associations between alpha diversity and muscle status; some studies reported reduced alpha diversity in sarcopenic participants, while others found no difference, but did identify specific microbiome composition signatures (e.g., *Prevotella*/*Bacteroides* ratio, *Coprococcus* and Lachnospiraceae abundance) that could differentiate individuals with sarcopenia from those with normal muscle status [51]. Overall, the data point toward a consistent relationship between reduced microbial diversity, loss of short-chain fatty acid-producing bacteria, and poorer skeletal muscle outcomes across different populations and age groups. However, because these studies are mainly cross-sectional, they cannot establish whether changes in the gut microbiome cause muscle loss or simply occur alongside it. Interventional studies would be needed to address this question.

### 7.2. Interventional Studies in Humans

Interventional studies have focused on the use of prebiotics and probiotics, exercise interventions, and/or dietary manipulation. Interventional evidence for probiotic and prebiotic supplementation on muscle outcomes has grown substantially in recent years, though results remain mixed in magnitude. A 2023 systematic review and meta-analysis of randomized controlled trials found that probiotic supplementation significantly improved muscle mass and global muscle strength in adults compared to placebo, though the authors noted considerable heterogeneity across studies and called for standardization of probiotic strains and dosing protocols in future trials [96]. A more recent meta-analysis focused specifically on older adults found that probiotic supplementation improved muscle strength, physical performance, and function, and suggested a beneficial, though less consistent, effect on muscle mass; prebiotic supplementation alone showed effectiveness for muscle strength specifically [97]. Beyond traditional probiotics, postbiotic and microbial-metabolite-based interventions have also shown promise: a randomized clinical trial found that supplementation with urolithin A, a gut microbiota-derived metabolite of ellagitannins, improved muscle endurance and mitochondrial health markers in older adults compared to placebo, illustrating that intervening on microbial metabolite availability directly, rather than on live bacterial strains, may represent an alternative therapeutic route within the gut–muscle axis [84].

Exercise interventions provide some of the clearest human evidence that muscle activity can reshape gut microbial composition, though the direction of this relationship and its practical significance for muscle outcomes specifically remain active areas of investigation. In a controlled trial, six weeks of endurance exercise training altered gut microbiota composition and function in both lean and obese human participants, with changes partially reversing after a subsequent sedentary washout period, indicating that exercise-induced microbiota shifts are meaningful but require sustained activity to persist [85]. Similarly, an exercise intervention among older, sedentary adults produced measurable changes in stool microbiota composition and metabolite profiles, suggesting that microbial responsiveness to exercise persists even in aging populations, a group that is vulnerable to sarcopenia [86]. In a human intervention study of men with prediabetes, the degree to which an individual’s gut microbiota could ferment dietary substrates into beneficial metabolites predicted how effectively exercise improved glucose homeostasis and insulin sensitivity, suggesting the microbiome does not merely respond to exercise passively but actively shapes an individual’s metabolic response to it [34]. However, a rapid review focused specifically on resistance training found that most included studies reported no significant change in overall gut microbiota taxonomic composition or diversity, in contrast to the more consistent findings from aerobic/endurance exercise trials, suggesting that exercise modality may meaningfully influence whether and how the gut microbiome responds [6].

Compared to probiotic and exercise interventions, direct human trials linking dietary fiber intake to skeletal muscle outcomes remain notably sparse, representing a clear gap in the interventional literature. The most relevant available human evidence comes from a controlled dietary intervention study that compared a high-fiber diet to a diet high in fermented foods; while the study’s primary outcomes centered on immune status and inflammatory markers rather than muscle mass or function directly, the high-fiber diet increased the community-level ability of participants’ gut microbiota to degrade fiber and produced individualized, rather than uniform, increases in short-chain fatty acid production across participants [98]. This individualized SCFA response is a meaningful consideration for gut–muscle axis research, specifically, since the muscle-mass associations described earlier in this paper depend on adequate microbial butyrate synthesis capacity [30], and a fiber intervention’s effectiveness for muscle outcomes may therefore depend heavily on an individual’s baseline microbiome composition rather than fiber intake alone. Future interventional trials that pair dietary fiber supplementation directly with muscle mass, strength, and performance outcomes, rather than inferring muscle relevance from metabolic or immune endpoints, are needed to establish this pathway with the same level of human evidence currently available for probiotics and exercise.

## 8. Clinical and Translational Implications

**Figure 2:**
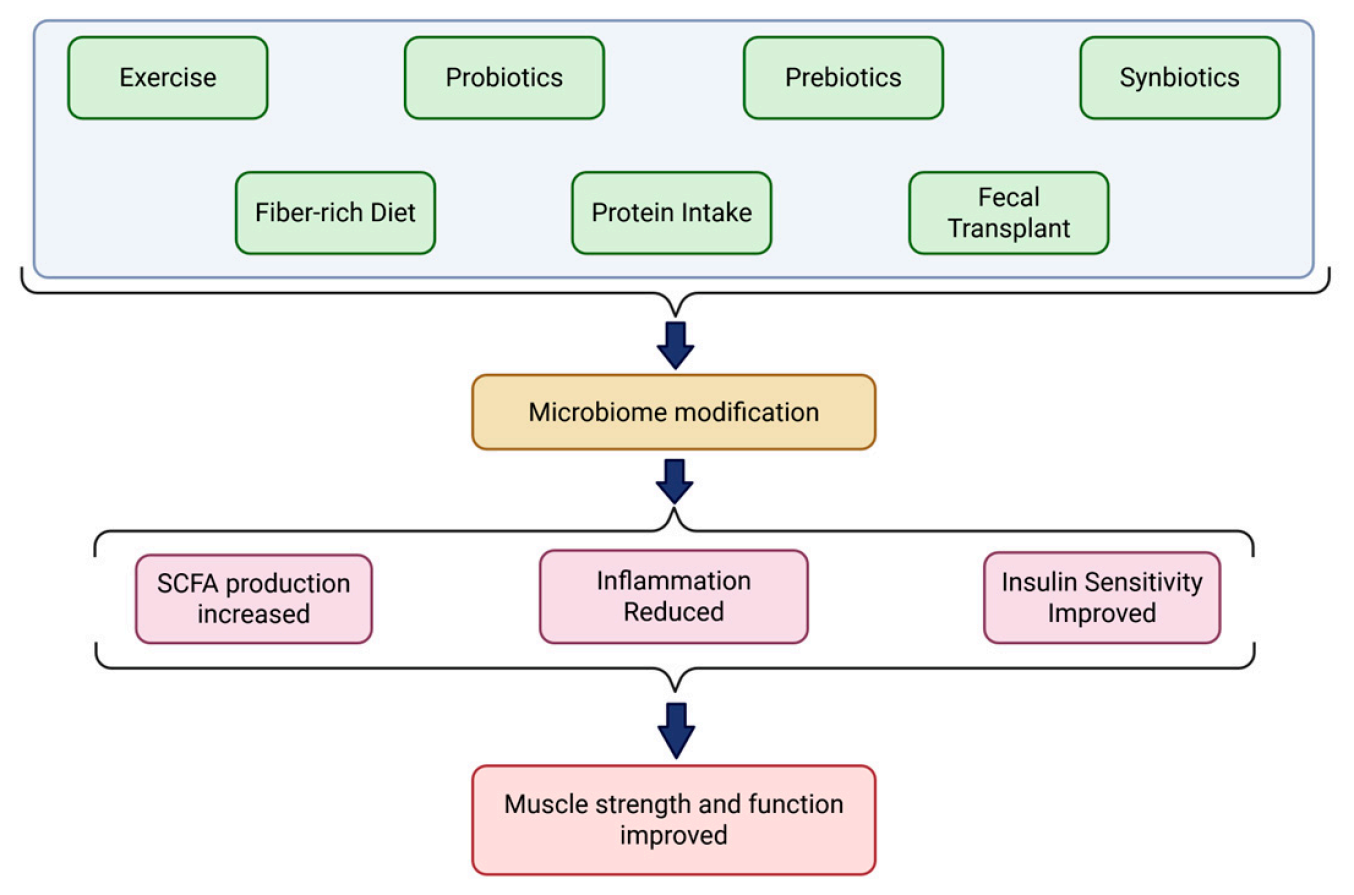
Exercise, dietary modification, probiotics, prebiotics, synbiotics, protein optimization, and fecal microbiota transplantation may beneficially alter gut microbial composition and function. These interventions may increase microbial diversity and SCFA production while reducing dysbiosis-associated inflammation. Improvements in gut barrier integrity, insulin sensitivity, mitochondrial function, and immune regulation support greater muscle strength, physical performance, and healthy aging, potentially reducing sarcopenia risk. Created in BioRender, Lauri O. Byerley (2026), https://BioRender.com.

Having established the mechanistic pathways and supporting evidence linking the gut microbiome to skeletal muscle, this section turns to their practical application (Figure 2). It first considers potential therapeutic strategies for targeting the gut microbiome to improve muscle function, then examines the gut–muscle axis specifically as a translational target: from microbiome-directed interventions such as probiotics, prebiotics, and synbiotics, and fecal microbiota transplantation, to current lifestyle-based strategies, emerging therapeutic approaches, and the research directions still needed to move this evidence from the laboratory into clinical practice.

### 8.1. Potential Therapeutic Strategies to Target the Gut Microbiome to Improve Muscle Function

Targeted modulation of the human gut microbiome is emerging as a potential therapeutic strategy to influence skeletal muscle metabolism, functional capacity, and age-related muscle decline [45,87,93]. From a translational perspective, microbiome-directed interventions are therapeutic approaches that can influence systemic metabolism, mitochondrial function, and inflammatory signaling pathways relevant to skeletal muscle physiology. Experimental and clinical studies suggest that microbiome-targeted strategies may influence muscle performance by reducing systemic inflammation, modulating metabolic signaling pathways, and improving mitochondrial efficiency, as observed in both animal and human studies [34,85].

Current research supports several microbiome-modulating intervention strategies, including probiotic supplementation with selected microbial strains, prebiotic dietary modulation, postbiotic metabolite enhancement, dietary pattern manipulation, and exercise-induced changes in gut microbial composition [85,98,99]. These emerging therapeutic strategies highlight multiple potential entry points for clinical modulation of the gut–muscle axis and provide a framework for microbiome-targeted interventions to preserve muscle health, functional capacity, and muscular performance.

### 8.2. Targeting the Gut–Muscle Axis with Microbiome-Targeted Intervention Strategies

#### 8.2.1. Current Lifestyle Strategies

Several lifestyle and nutritional interventions already implemented in clinical and public health settings have been shown to influence the composition and activity of the gut microbiome. Among these, dietary modification is one of the most important determinants of microbial diversity and metabolic output. Diets rich in fermentable substrates, such as dietary fiber, promote microbial fermentation and increase the production of metabolites, which play important roles in metabolic regulation, immune signaling, and mitochondrial function relevant to skeletal muscle physiology [98,100]. These metabolites are linked to improvements in metabolic homeostasis and reductions in systemic inflammation, both of which influence muscle metabolism and function [24,30].

Physical activity also plays an important role in modulating the gut microbiome. Studies in athletes and previously sedentary individuals demonstrate that exercise can increase microbial diversity and alter the abundance of metabolically active bacterial taxa, thereby improving metabolic health [85,86,101]. In addition, emerging work suggests that microbial metabolism may interact with exercise physiology through mechanisms such as lactate metabolism and SCFA production, potentially influencing endurance capacity and metabolic efficiency [102,103].

Probiotic supplementation is also widely used as a strategy to introduce beneficial microorganisms capable of modifying microbial community composition and metabolic activity. Clinical and translational studies have demonstrated that probiotic and synbiotic supplementation may improve metabolic biomarkers, inflammatory signaling, and host physiological responses in conditions associated with metabolic dysfunction [104,105,106]. Collectively, these findings demonstrate that lifestyle and nutritional interventions can modify the gut microbiome, thereby influencing systemic metabolic pathways relevant to skeletal muscle physiology. However, these approaches remain relatively nonspecific, and emerging research suggests that more targeted microbiome-directed therapies may allow more precise modulation of host–microbe interactions.

#### 8.2.2. Probiotic Supplementation

Probiotic supplementation is among the most widely studied strategies for modulating the gut microbiome and may influence skeletal muscle physiology via microbial and metabolic pathways. Probiotics are live microorganisms that confer health benefits to the host when administered in adequate amounts and are commonly used to alter microbial composition and metabolic activity in the gastrointestinal tract [107]. Experimental and translational studies suggest that probiotic-induced shifts in microbial communities can influence host metabolic signaling, inflammatory pathways, and microbial metabolite production, all of which may affect skeletal muscle metabolism and systemic energy regulation [21,24,108].

Several probiotic organisms, including *Lactobacillus* and *Bifidobacterium* species, have been investigated for their potential to influence microbial metabolite production, inflammatory signaling, and metabolic homeostasis in skeletal muscle physiology. Clinical studies have also demonstrated improvements in metabolic biomarkers and inflammatory profiles following probiotic or synbiotic supplementation in individuals with obesity, metabolic disease, and related conditions [104,105]. Importantly, probiotic effects are strain-specific, and observed physiological outcomes depend on the particular microbial strain, dosage, and host context. Consequently, ongoing research evaluates targeted probiotic interventions as potential strategies for microbiome modulation and metabolic regulation within the broader framework of the gut–muscle axis.

#### 8.2.3. Prebiotics and Synbiotics

Prebiotic and synbiotic interventions represent complementary strategies for modulating the gut microbiome by promoting the growth and metabolic activity of beneficial microbial populations. Prebiotics are non-digestible dietary substrates that selectively stimulate the growth of specific microorganisms within the gastrointestinal tract, most commonly fermentable dietary fibers that support microbial metabolism and influence host metabolic regulation, inflammatory signaling, and mitochondrial function relevant to skeletal muscle physiology [24,30].

Synbiotics combine probiotic microorganisms with prebiotic substrates designed to enhance microbial colonization and metabolic activity, potentially amplifying beneficial microbiome-mediated effects. Clinical studies have demonstrated that synbiotic supplementation can improve metabolic biomarkers and inflammatory profiles in individuals with obesity and metabolic disorders, suggesting broader systemic effects of microbiome modulation [104,105].

Together, these approaches illustrate how targeted nutritional substrates and combined microbial interventions may influence microbial metabolite production and metabolic signaling pathways within the emerging framework of the gut–muscle axis.

#### 8.2.4. Fecal Microbiota Transplantation

Fecal microbiota transplantation (FMT) is a direct method for modifying gut microbial communities by transferring intestinal microbiota from a healthy donor to a recipient. While currently established as a therapy for recurrent *Clostridioides difficile* infection, FMT has the potential to influence host metabolic regulation via restoration of microbial diversity and metabolic activity. Transplantation of a metabolically favorable microbiome can alter host physiology through increased production of microbial metabolites, modulation of bile acid metabolism, and improved intestinal barrier integrity, all of which influence systemic inflammatory signaling and metabolic homeostasis [21,24,108]. While there is no direct evidence that FMT improves skeletal muscle functional outcomes in humans, it could contribute to improved mitochondrial function, enhanced substrate utilization, and reduced chronic inflammation.

Clinical trials further support these mechanistic pathways. For example, transplantation of microbiota from lean donors has been shown to improve insulin sensitivity in individuals with metabolic syndrome, an effect associated with shifts in microbial composition and increased production of beneficial microbial metabolites [109]. Similarly, randomized controlled trials in individuals with obesity have demonstrated that FMT can modify microbial community structure and influence metabolic biomarkers, particularly when combined with dietary interventions that support microbial fermentation [110,111]. These findings suggest that large-scale restructuring of the gut microbiome may influence host metabolic and inflammatory pathways relevant to skeletal muscle physiology. Although FMT remains primarily an experimental intervention outside of infectious disease treatment, ongoing research continues to explore its potential applications in metabolic disorders, aging, and conditions associated with muscle dysfunction within the emerging framework of the gut–muscle axis [34,94].

#### 8.2.5. Therapeutic Translation of the Gut–Muscle Axis

The intervention strategies discussed in the preceding section illustrate several pathways of modulating the gut microbiome to influence host physiology. Approaches including probiotic supplementation, dietary modification, exercise-induced changes in the microbiome, and fecal microbiota transplantation demonstrate that altering microbial communities and their metabolic outputs is possible through both lifestyle and targeted interventions. Increasing evidence suggests that these microbiome-mediated changes influence host metabolic regulation, inflammatory signaling, and mitochondrial function, processes that play important roles in skeletal muscle physiology [21,24,87]. Building on this mechanistic framework, the following section examines how these findings inform current strategies, emerging microbiome-targeted therapies, and future approaches aimed at modulating the gut–muscle axis to support skeletal muscle health.

#### 8.2.6. Emerging Therapeutic Approaches

Advances in microbiome science have led to the development of new strategies aimed at targeted manipulation of microbial communities and their metabolic outputs. Synbiotic formulations, which combine probiotic organisms with fermentable substrates that promote their growth, represent one such approach designed to enhance microbial colonization and metabolic activity [105,112]. These interventions aim to optimize microbial metabolic pathways that support beneficial host outcomes, including improved metabolic regulation and reduced inflammatory signaling.

In parallel, increasing attention has focused on microbial metabolites themselves as therapeutic mediators. Microbiome-derived compounds/metabolites influence host signaling pathways involved in immune regulation, energy metabolism, and mitochondrial function [24,113]. These pathways are particularly relevant to skeletal muscle physiology because mitochondrial health, inflammatory signaling, and substrate utilization play central roles in muscle maintenance and metabolic adaptation. For example, microbial fermentation products may influence exercise-related metabolic responses and the physiological adaptations associated with physical activity [34,63].

Additional strategies include microbiome-directed nutritional interventions designed to selectively promote microbial taxa associated with favorable metabolic and physiological outcomes. Controlled dietary interventions have demonstrated that altering macronutrient composition can rapidly reshape microbial metabolic activity and host metabolic signaling [100,114]. Together, these approaches represent a shift from broad microbiome modulation toward targeted manipulation of microbial metabolic pathways that influence host physiology.

## 9. Limitations of Current Research and Gaps in Knowledge

### 9.1. Dietary and Nutritional Determinants

Several diet- and nutrition-related limitations hamper this research, including challenges in assessing dietary intake, designing interventions, and conducting controlled feeding studies. Despite abundant data showing that fiber intake, protein source, and overall dietary pattern each independently shape microbial composition and muscle outcomes, many observational studies linking gut microbiota composition to muscle mass or function do not adequately account for habitual dietary intake. Without rigorous dietary assessment, it remains difficult to determine whether the microbiome exerts an independent effect on muscle physiology or primarily serves as a downstream biomarker of dietary quality.

The design of intervention studies also limits our understanding of the gut microbiome’s role in skeletal muscle function. Fiber and prebiotic intervention studies vary considerably in dose, fiber subtype, formulation, and duration. A systematic review of dietary fiber interventions found that although fiber intake affects microbiota composition and derived metabolites, it does not produce a consistent, significant increase in SCFA levels across studies in healthy adults. This underscores that fiber-type heterogeneity, rather than a single well-characterized exposure, currently limits cross-study comparability and the ability to derive standardized dietary recommendations.

Much of the human evidence in this field derives from cross-sectional cohorts or short-duration supplementation trials rather than controlled feeding studies with verified nutrient intake. This limits the ability to establish dose–response relationships between specific dietary components and gut–muscle axis outcomes. For example, a combination of plant- and animal-based proteins has recently been proposed as more favorable for gut microbiota eubiosis and muscle protein synthesis than reliance on a single protein source, such as red meat. Few studies in this literature systematically report or control for protein source, quantity, and timing simultaneously. Given that the balance, type, and amount of dietary protein can substantially affect intestinal microbiota composition, this represents a meaningful source of unexplained heterogeneity across studies. dies.

Finally, the gut microbiota extensively biotransforms dietary polyphenols into bioactive compounds that vary with the composition and metabolic function of an individual’s gut bacterial community. Hence, polyphenol interventions that appear effective in one population or individual may not generalize. Current study designs rarely address this limitation through pre-intervention microbiome stratification.

### 9.2. Heterogeneity

One limitation is substantial heterogeneity in study design, population characteristics, and methodology, which complicates direct comparisons across studies and weakens the strength of any meta-analytic conclusions. In particular, systematic reviews examining the gut microbiome’s relationship with muscle mass, strength, and function have noted heterogeneity in participant age, sex, health status, and the specific muscle outcome measures [51,58]. The lack of standardized methods for characterizing the gut microbiome also compounds the problem. Studies vary in what they report. For example, some report relative abundance, absolute counts, functional metagenomic capacity, or serum metabolite concentrations, and even studies measuring the same metabolite, such as butyrate, differ in whether they assess fecal, serum, or microbial synthesis-capacity levels [30]. A parallel problem exists on the muscle side. There is a lack of consensus on whether muscle mass, muscle strength, or physical performance is the most meaningful outcome, and studies often report only one or two of these three components. For sarcopenia, studies often do not use the full sarcopenia phenotype described in current clinical consensus definitions [5]. Two studies illustrate this point. A separate meta-analysis of probiotic-fortified product trials in older adults reported considerably higher statistical heterogeneity (I^2^ = 89.5% for muscle mass and I^2^ = 89.9% for muscle strength) than Prokopidis et al. (2023), whose outcomes ranged from I^2^ = 0% to 64%, illustrating that heterogeneity estimates themselves vary substantially depending on which trials are pooled and how outcomes are defined [96,115].

### 9.3. Study Design

A second major gap is the field’s continued reliance on cross-sectional, observational, and animal-model evidence, which cannot establish the direction or causality of the relationships described throughout this paper. Much of the mechanistic evidence connecting SCFA production, gut permeability, and inflammatory signaling to muscle atrophy pathways originates from animal models or in vitro cell culture systems. It is unclear how directly these mechanisms translate to human physiology [44]. Even where human data exist, most studies are correlational, making it difficult to determine whether gut microbial composition drives changes in muscle mass and function or is instead a downstream consequence of muscle-related lifestyle factors such as diet and physical activity [88,90]. Notably, one of the few studies in this literature to incorporate causal inference, using a one-sample Mendelian randomization approach, found only a modest effect size despite reaching statistical significance, underscoring both the promise and the current limits of moving beyond simple correlation [30]. Longitudinal studies that track microbiome composition and muscle outcomes over extended timeframes, alongside interventional trials that directly manipulate the microbiome, such as through targeted probiotic strains, prebiotic fiber formulations, or fecal microbiota transplantation, while simultaneously measuring standardized muscle mass, strength, and performance endpoints, represent the clearest path toward resolving these open questions and translating the gut–muscle axis from an area of mechanistic interest into one of clinical application.

## 10. Future Directions

As this review shows, skeletal muscle function relies on the interaction between dietary intake of specific nutrients and their fermentation/putrefaction in the colon. Future studies must consider this interdependence and keep in mind that it is bidirectional.

As for diet, future studies should prioritize longer-duration designs that connect dietary/nutraceutical modulation of the gut microbiome to these functional and clinical outcomes, particularly in aging populations where sarcopenia prevention has direct clinical significance. More controlled feeding studies, rather than free-living supplementation trials, are needed with the aim of developing personalized nutrition. For example, with future human trials and observational cohorts, the protein source (plant vs. animal), total quantity relative to body weight, and distribution across meals should be consistently reported. For fiber, future research should investigate microbiome-stratified or personalized nutrition approaches, like pre-intervention microbial profiling to identify individuals likely to respond to a given prebiotic, polyphenol, or synbiotic regimen.

For the gut microbiome, future research should focus on identifying targeted strategies to modulate it and support skeletal muscle health across diverse populations and physiological conditions. Advances in sequencing technologies and multi-omics approaches have substantially improved the ability to characterize microbial community composition and functional metabolic outputs [115]. These technologies are increasingly combined with metabolomic and systems biology approaches to better understand host–microbe interactions that influence metabolic and physiological outcomes.

This growing mechanistic understanding may enable the development of personalized nutrition/microbiome medicine, in which specific microbial taxa or metabolite profiles, such as butyrate synthesis capacity or the abundance of species like *Faecalibacterium prausnitzii*, serve as biomarkers to identify individuals at elevated risk of muscle mass or strength decline before clinical symptoms of sarcopenia emerge [94]. Realizing this potential, however, will require sustained interdisciplinary collaboration among microbiologists, exercise physiologists, and nutrition scientists, since no single field currently possesses the full expertise needed to translate microbial biomarker discovery into validated clinical tools; microbiologists are needed to characterize functionally relevant taxa and metabolic pathways, physiologists to establish how these microbial signals translate into measurable changes in muscle physiology, and nutrition scientists to design dietary and supplementation strategies capable of shifting microbial composition in a targeted, reproducible way. Translational studies bridging these disciplines will also be necessary to clarify the efficacy, safety, and long-term effects of microbiome-targeted therapies in clinical and population health contexts. Emerging research exploring microbial metabolites, targeted probiotic strains, and microbiome-directed nutritional strategies suggests that microbiome modulation may eventually become an important component of strategies aimed at preserving skeletal muscle mass, metabolic function, and physical performance throughout aging [21,87,93].

Taken together, these developments indicate that the gut microbiome represents a critical interface between environmental exposures, host metabolism, and skeletal muscle physiology. As mechanistic understanding advances through coordinated efforts across microbiology, physiology, and nutrition science, microbiome-directed interventions may offer new opportunities to clinically influence muscle health and metabolic function across the lifespan.

## 11. Conclusions

This review has examined emerging evidence for a gut–muscle axis, focusing on microbial metabolites, many derived from dietary sources, that mediate this relationship. Diet is the primary modifiable factor governing gut microbial composition and metabolic output. Gut microbial composition and metabolic activity (e.g., short-chain fatty acid production, amino acid metabolism, immune modulation, gut barrier integrity, and endocrine signaling) are key mechanistic outputs that shape skeletal muscle mass, strength, and function. These metabolites act through interconnected pathways to influence the anabolic–catabolic balance governing muscle tissue. Taken together, the evidence supports the gut–muscle axis as a biologically plausible and increasingly well-characterized pathway linking gut microbial metabolite production to muscle health. However, significant limitations remain, including methodological inconsistencies, a shortage of standardized muscle and microbiome metrics, and a persistent reliance on correlational rather than causal human data. Addressing these gaps will require longitudinal, interdisciplinary, and well-controlled intervention studies that pair microbiome-targeted strategies with rigorous assessments of muscle outcomes and dietary intake. If that work bears out, the gut microbiome may serve not only as a mechanistic contributor to muscle health but also as a clinically actionable target for preserving muscle mass, strength, and physical function across aging. More broadly, the gut–muscle axis reframes skeletal muscle health as an outcome shaped in part by microbial metabolic activity, not exercise and protein intake alone. As precision and personalized nutrition approaches continue to evolve, they may offer novel, metabolite-targeted opportunities to preserve muscle mass, strength, and functional capacity across the lifespan, positioning the gut microbiome as a central and clinically actionable lever for intervention in this axis.

## Figures and Tables

**Table 1:** Microbial metabolites implicated in skeletal muscle function.

Metabolite Class	Examples	Produced from	Effects on Skeletal Muscle
SCFAs	Acetate, Propionate, Butyrate	Fermentation of dietary fiber by gut bacteria	Enhance mitochondrial function, improve insulin sensitivity, increase glucose utilization, reduce inflammation, support muscle protein synthesis, and may improve endurance performance. Butyrate appears especially beneficial for preserving muscle mass during aging [24,25,26].
Lactate-Derived Cross-Feeding Metabolites	SCFAs generated from lactate-utilizing bacteria	Extreme exercise-induced lactate is metabolized by gut microbes	Improve energy efficiency, exercise adaptation, and endurance capacity [24,26].
BCAA-Related Metabolites	Leucine-, isoleucine-, and valine-derived metabolites	Microbial synthesis and metabolism of amino acids	Support mTOR signaling, muscle protein synthesis, muscle growth, repair, and recovery [24,25].
Secondary Bile Acids	Deoxycholic acid (DCA), Lithocholic acid (LCA)	Bacterial transformation of primary bile acids	Regulate muscle metabolism through FXR and TGR5 signaling pathways, influence energy expenditure, mitochondrial activity, and maintenance of muscle mass and function [27,28].
Indole Derivatives	Indole, Indole-3-propionic acid (IPA), Indole-3-acetic acid (IAA)	Bacterial metabolism of tryptophan	Reduce systemic inflammation, improve gut barrier integrity, and may protect against muscle loss associated with aging and chronic disease [25,28].
Polyamines	Spermidine, Spermine, Putrescine	Produced by microbes and host metabolism	Promote cellular growth, autophagy, mitochondrial quality control, and maintenance of muscle mass during aging [24,25].
Phenolic & Other Signaling Metabolites	Various phenols and microbial signaling compounds	Metabolism of dietary polyphenols and other nutrients	Influence inflammation, oxidative stress, endocrine signaling, and insulin/IGF-1 pathways that affect muscle function [24,25].

**Table 2:** Representative human observational and interventional studies examining the association between gut microbiome composition/function and skeletal muscle mass, strength, or performance. Studies are drawn from the broader literature cited in this review; meta-analyses and systematic reviews are excluded to highlight primary data sources.

Study	Design	Population (*N*)	Microbiome Measure	Muscle/Function Outcome	Key Finding	Ref.
Observational Studies						
Lv et al., 2021	Cross-sectional + one-/two-sample Mendelian randomization	Healthy menopausal women (*N* = 482)	Shotgun metagenomics + serum SCFA + GWAS	DXA-derived skeletal muscle mass, appendicular lean mass	Causal effect of gut microbial butyrate synthesis on appendicular lean mass (β = 0.04, *p* = 0.003); one of the few studies applying causal inference	[30]
Grahnemo et al., 2023 (HUNT study)	Population-based cohort	Large Norwegian cohort	16S rRNA sequencing + GWAS	Appendicular lean mass	Identified 3 bacterial species associated with increased appendicular lean mass	[76]
Sakaguchi et al., 2025 (Wakayama Study)	Cross-sectional	Community-dwelling older Japanese adults	16S rRNA sequencing	Skeletal muscle mass	Genus *Blautia* associated with reduced muscle mass	[77]
Wang et al., 2022 (Xiangya Sarcopenia Study)	Cross-sectional	Adults ≥ 50 (*N* = 1417), China	Shotgun metagenomics	Grip strength, SPPB, Chair Stand Test, gait speed, BIA	Sarcopenic participants had significantly higher abundance of 7 species (e.g., *Desulfovibrio piger*), correlated with sarcopenia severity	[78]
Han et al., 2022	Cross-sectional	Adults ≥ 65 (*N* = 88), Taiwan	16S rRNA sequencing + fecal butyrate	Grip strength, gait speed, Sit-and-Reach Test, BIA	Fecal butyrate significantly correlated with skeletal muscle mass; reduced butyrate-producers in low-muscle-mass group	[79]
Ticinesi et al., 2020	Cross-sectional (proof-of-concept)	Adults ≥ 70 (*N* = 17), Italy	Shotgun metagenomics	SPPB, grip strength, gait speed, Chair Stand Test, BIA	*F. prausnitzii* five-fold higher in normal vs. low muscle-function group	[80]
Cox et al., 2021	Cross-sectional	Adults ≥ 65 (*N* = 204), UK	16S rRNA sequencing	Chair Stand Test, DXA	Poor appetite associated with altered microbiome diversity/composition and reduced muscle strength	[81]
Picca et al., 2019 (BIOSPHERE study)	Cross-sectional	Adults ≥ 70 (*N* = 35), Italy	16S rRNA sequencing	SPPB, 400 m Walk Test, DXA	Distinct microbial, inflammatory, and metabolic signatures in physical frailty/sarcopenia	[82]
Interventional Studies						
Tominaga et al., 2021	Single-arm cohort	Sarcopenic adults ≥ 82 (*N* = 6), Japan	1-kestose prebiotic, 12 weeks	SMI, SMM, TMM, grip strength, gait speed	↑ *Bifidobacterium longum*; significant improvements in muscle mass measures after 12 weeks	[83]
Liu et al., 2022 (JAMA Netw Open)	RCT	Older adults	Urolithin A supplementation	Muscle endurance, mitochondrial health	Improved muscle endurance and mitochondrial biomarkers vs. placebo	[84]
Allen et al., 2018	Exercise intervention (non-randomized)	Lean and obese adults	6-week endurance exercise training	Gut microbiota composition/function	Exercise-induced compositional and functional microbiota shifts, partially reversed after training cessation	[85]
Erlandson et al., 2021	Single-arm exercise intervention	Sedentary adults 50–75 y (*N* = 22)	24-week combined aerobic + resistance exercise	Stool microbiota, SCFA metabolites	↑ *Bifidobacterium*, *Oscillospira*, *Anaerostipes*; ↓ *Prevotella*, *Oribacterium*	[86]

Abbreviations: BIA, bioelectrical impedance analysis; DXA, dual-energy X-ray absorptiometry; GWAS, genome-wide association study; RCT, randomized controlled trial; SCFA, short-chain fatty acid; SMI, skeletal muscle index; SMM, skeletal muscle mass; SPPB, Short Physical Performance Battery; TMM, total muscle mass; ↑, increase, ↓, decrease.

## Data Availability

Questions on the content of this paper should be addressed to the corresponding author. All data is presented in the tables.
